# *In silico* prediction of cytotoxic T-cell epitopes from *Helicobacter pylori* virulence factors using an immunoinformatics approach

**DOI:** 10.5114/bta/208778

**Published:** 2025-09-22

**Authors:** Demy Valerie Chacon, Kiana Alika Co, Daphne Noreen Enriquez, Aubrey Love Labarda, Reanne Eden Manongsong, Edward Kevin Bragais

**Affiliations:** 1 Department of Biology, School of Science and Engineering, Ateneo de Manila University, Quezon City, Philippines; 2 Department of Pharmaceutical Chemistry, College of Pharmacy, University of the Philippines Manila, Manila, Philippines

**Keywords:** immunoinformatics, *Helicobacter pylori*, cytotoxic T lymphocytes, epitopes

## Abstract

**Background:**

*Helicobacter pylori* infects approximately half of the global population, leading to gastric and duodenal ulcers. Despite the availability of antibiotics, challenges such as patient reluctance, high treatment costs, and antibiotic resistance limit their effectiveness, making vaccination a promising alternative. This study used immunoinformatics to identify candidate epitopes for a multiepitope vaccine construct against *H. pylori*.

**Materials and methods:**

The protein variability server was utilized for conservation analysis. The epitopes were screened for antigenicity, allergenicity, toxicity, cross-reactivity, and population coverage. Selected epitopes were docked with their corresponding human leukocyte antigen (HLA) alleles, and thermodynamic quantities were determined. Five virulence factors – HopZ, SabA, HP-NAP, OipA, and urease – were selected for their critical roles in bacterial adhesion, immune modulation, and stress survival.

**Results:**

Conservation analysis revealed a highly conserved protein sequence (Shannon index ≤ 0.1). The predicted epitopes had an IC_50_ value of ≤ 500 nM, indicating strong binding to the corresponding HLAs, with an estimated population coverage of more than 90% in the Southeast Asian region. The predicted epitopes were identified as probable nonallergens, nontoxic, and noncross-reactive (*E* value >1.0). Molecular docking analysis showed that the candidate epitopes could bind strongly and spontaneously with their corresponding HLA proteins, as evidenced by low negative Gibbs free energy (ΔG) values and dissociation constants (*K*_D_ < 100 nM).

**Conclusion:**

The epitopes predicted from the five virulence factors present promising candidates for future *H. pylori* vaccine design. Further *in vitro* and *in vivo* experiments are recommended to validate these preliminary findings.

## Introduction

*Helicobacter pylori* is a microaerophilic, spiral-shaped, Gram-negative bacterium that predominantly colonizes the human gastric mucosa. It is genetically diverse, with strains exhibiting variations in virulence factors (VFs) that influence the severity of infection and associated pathological outcomes (Muńoz et al. 2020). *H. pylori*’s ability to adapt to the harsh acidic environment of the stomach, along with its genetic variability, contributes to a wide range of immune responses and complications, including gastritis, peptic ulcers, and an increased risk of gastric cancer (Sharndama and Mba 2022; Ali and AlHussaini 2024). Given its classification as a Class 1 carcinogen by the World Health Organization, the long-term persistence of *H. pylori* in the human stomach has a significant impact on public health worldwide (Sharndama and Mba 2022).

The global burden of *H. pylori* infection is substantial, affecting approximately 63.4% of the global population, with notably high prevalence rates in Southeast Asia (43.1%) and the Philippines (53.7%) (Sollano 2015; Kharel et al. 2020). In particular, infection is strongly associated with gastric (64.4%) and duodenal ulcers in affected individuals (Schöttker et al. 2012). Despite the availability of standard antibiotic-based therapies – such as triple therapy with proton pump inhibitors (PPIs) and antibiotics like amoxicillin and clarithromycin – the rising incidence of antibiotic-resistant strains, coupled with issues such as poor patient compliance, limits the long-term efficacy of these treatments. These challenges underscore the urgent need for alternative therapeutic strategies, particularly preventive measures such as vaccine development (Kim et al. 2015).

One promising approach to addressing this problem is the application of immunoinformatics, which integrates computational methods with immunology to identify conserved and immunogenic epitopes from *H. pylori*’s virulence factors (Bragais et al. 2025). Immunoinformatics enables the rational design of peptide-based vaccines, offering broad protection without the risk of resistance, and presents a viable alternative to conventional antibiotic therapy. Specifically, the field employs computational tools to predict antigenic epitopes – regions of a pathogen that stimulate an immune response – thereby facilitating vaccine development more efficiently and cost-effectively, without the need for extensive experimental trials (Mortazavi et al. 2024).

In this study, five key virulence factors – *H. pylori* neutrophil-activating protein (HP-NAP), outer inflammatory protein A (OipA), sialic acid-binding adhesin (SabA), outer membrane protein (HopZ), and urease were selected for their critical roles in *H. pylori* pathogenesis and their potential as immunogenic targets for vaccine development. These virulence factors are involved in essential processes such as immune modulation, bacterial adherence, and stress survival (Sedarat and Taylor-Robinson 2024).

HP-NAP and OipA play significant roles in immune modulation. HP-NAP stimulates neutrophils to generate reactive oxygen species (ROS), contributing to inflammation and protecting *H. pylori* from oxidative stress, thereby enhancing its survival under immune attack (Satin et al. 2000; Fu 2014). OipA, which induces inflammatory responses and facilitates bacterial adhesion to host cells, is associated with an increased likelihood of gastric cancer and peptic ulcer (Al-Maleki et al. 2017). The involvement of HP-NAP and OipA in immune evasion and inflammation underscores their importance as vaccine targets.

SabA and HopZ are critical for *H. pylori* adherence to host tissues, a key factor in chronic infection and disease progression. SabA binds to sialylated carbohydrates in human cells, facilitating bacterial colonization and contributing to chronic inflammation – a process implicated in gastric cancer (Sedarat and Taylor-Robinson 2024). Its phase-variable expression enables *H. pylori* to adapt to diverse inflammatory environments. Similarly, HopZ mediates bacterial attachment to gastric epithelial cells, and its phase-variable expression enhances *H. pylori*’s ability to evade the immune system, thereby promoting pathogenicity (Sedarat and Taylor-Robinson 2024; Alzahrani et al. 2014).

Urease, an essential virulence factor in *H. pylori* pathogenesis, neutralizes gastric acid and enables the bacterium to survive in the highly acidic stomach environment (Kusters et al. 2006). It also modulates the host immune response, contributing to the persistence of *H. pylori* and promoting ulcer formation as well as gastric cancer progression (Sedarat and Taylor-Robinson 2024). Urease’s broad impact on bacterial survival and immune evasion underscores its importance as a critical target for vaccine development.

These selected VFs are highly conserved across *H. pylori* strains, ensuring that a vaccine targeting them could provide broad protection against diverse isolates. The conservation of these factors enhances the likelihood of sustained vaccine efficacy across different strains (Elbehiry et al. 2025). Previous studies have shown that these VFs are promising candidates for immunization due to their essential roles in the bacterium’s survival and pathogenicity. Their conservation and functional significance in immune modulation, bacterial adherence, and acid resistance make them ideal targets for the development of a multiepitope vaccine (Osterloh 2022).

The growing importance of immunoinformatics in vaccine development has led to its widespread application, particularly in designing vaccines against complex pathogens such as *H. pylori*. Immunoinformatics applies computational methods to predict antigenic epitopes capable of triggering an immune response, thereby enabling more efficient vaccine design (Bragais et al. 2025; Mortazavi et al. 2024). For *H. pylori*, this approach is especially advantageous due to the bacterium’s genetic variability, immune evasion mechanisms, and antigenic diversity. By identifying conserved epitopes that are less prone to mutation, immunoinformatics supports the development of vaccines with broad efficacy across multiple *H. pylori* strains. Moreover, it facilitates the design of multiepitope vaccines targeting several virulence factors simultaneously, increasing the likelihood of eliciting a robust and durable immune response.

In this study, immunoinformatics tools were employed to predict T-cell epitopes from the five selected virulence factors of *H. pylori*. The objective is to design a multiepitope vaccine capable of stimulating a comprehensive immune response by targeting key processes essential for bacterial survival and immune modulation. By focusing on these critical virulence factors, this study aims to contribute to the development of a vaccine that offers a viable alternative to traditional antibiotic therapies, addressing the challenges posed by antibiotic resistance. The identified epitopes, predicted to provide broad population coverage and minimal adverse reactions, may serve as a foundation for future epitope-based vaccine designs.

## Materials and methods

### Retrieval of Helicobacter pylori protein sequences

**Figure 1:**
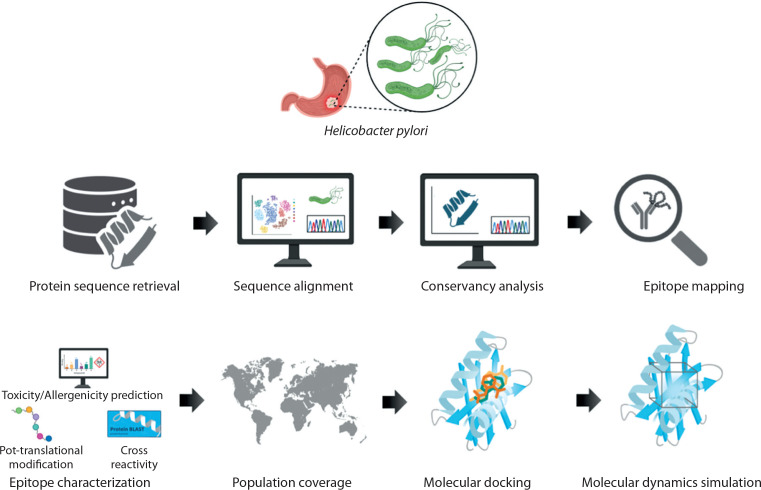
Workflow for virulence factor protein analysis of *Helicobacter pylori*

Five virulence factors of *H. pylori* – OipA, HpNap, SabA, HopZ, and Urease – were selected from the virulence factor database (VFDB) (https://www.mgc.ac.cn/VFs/main.htm). For each protein, more than 100 fulllength amino acid sequences were retrieved from the National Center for Biotechnology Information (NCBI) protein data bank (https://www.ncbi.nlm.nih.gov/protein). The workflow of this study is shown in Figure 1.

### Sequence alignment

Clustal Omega (https://www.ebi.ac.uk/Tools/msa/clustalo) was used to generate sequence alignments from the retrieved protein sequences. This software employs a full-distance matrix and the mBed method, which is efficient for handling large datasets and requires a minimum of 100 sequences for accurate clustering using the bisecting k-means algorithm (Sievers and Higgins, 2018). It produces a more accurate guide tree that determines the order of sequence comparisons and refines the final alignment (Blackshields et al. 2010).

### Conservation analysis

The aligned sequences for each virulence factor were uploaded to the Protein Variability Server (https://imed.med.ucm.es/PVS). Shannon entropy, a method for measuring sequence variability within a protein family, was selected for analysis. This method requires at least 100 sequences and provides a sensitive measure of protein family diversity (Protein Variability Server, n.d.). The analysis was performed using the consensus sequence – representing the most common amino acid at each position – as the reference (García-Boronat et al. 2008). The “mask sequence variability” option was applied with a variability threshold of 0.1 to identify highly conserved regions (low variability) suitable for further analysis (García-Boronat et al. 2008). Only sequences exceeding 11 amino acids in length were retained.

### Epitope mapping

The Proteasomal Cleavage/TAP Transport/MHC Class I Combined Predictor of the Immune Epitope Database (IEDB) was used to predict potential T-cell epitopes (http://tools.iedb.org/processing). NetMHCcons, a consensus method that integrates multiple prediction algorithms for MHC binding, was selected due to its higher reliability compared with individual prediction methods (Karosiene et al. 2012). Since *H. pylori* is a human pathogen, the retained sequences were evaluated against a set of human leukocyte antigens (HLA), which is provided in the supplementary material.

For the TAP transport efficiency prediction, the maximum precursor extension parameter was set to 1, meaning only the presented epitope and peptides with a single additional N-terminal residue were considered. The α parameter was set to 0.2, indicating that N-terminal residues contribute 20% as much as other positions to the overall TAP score (Peters et al. 2003; Fleri et al. 2017). Peptides were selected if they met the following criteria: a positive proteasome score, a positive TAP score, and an MHC-I binding score ≤ 500 nM. Peptides with positive proteasome and TAP scores are more likely to be generated, transported efficiently, and presented by MHC class I molecules (Peters et al. 2003). Those with strong MHC-I binding affinity (≤ 500 nM) are more likely to be recognized by cytotoxic T cells and elicit an immune response (Paul et al. 2013).

### Evaluation for post-translational modifications

The selected candidate epitopes were analyzed for potential post-translational modifications (PTMs) using the UniProt PTM/Processing database (https://www.uniprot.org). Only candidate epitopes without PTM regions were retained for subsequent toxicity and allergenicity prediction. Targeting non-PTM regions within conserved sequences is advantageous for vaccine design, as these regions are less likely to undergo modifications that could hinder immune recognition (Mahmud et al. 2021).

### Toxicity and allergenicity prediction

The nonPTM epitopes were evaluated for potential toxicity using the ToxinPred server (https://webs.iiitd.edu.in/raghava/toxinpred/). The Swiss-Prot-based SVM method was employed with a default *e*-value cut-off of 10, offering an estimated accuracy of 90% (Gupta et al. 2013). This setting identifies sequences with low toxicity potential while allowing those with *e*-values up to 10 to be considered (Rathore et al. 2023). Subsequently, AllergenFP 1.0 (https://ddg-pharmfac.net/AllergenFP/) was used to assess allergenicity. This tool applies the Tanimoto sequence score to compare the epitope with the nearest allergenic peptide in the probable allergen database (Dimitrov et al. 2013).

### Analysis of probable cross-reactivity

Potential cross-reactivity of the predicted epitopes was assessed using the Protein Basic Local Alignment Search Tool (BLAST) on the NCBI server (https://blast.ncbi.nlm.nih.gov.ph/BLAST.cgi?PAGE=Proteins). Searches were conducted against the standard nonredundant human protein sequence database to identify similarities with the human proteome. Epitopes showing minimal sequence homology to human proteins (*e*-value > 1) were prioritized to minimize the risk of cross-reactivity and potential autoimmune responses (Pearson 2013; Hauswedell et al. 2024).

### Calculation of population coverage

Population coverage analysis was performed using the IEDB tool (http://tools.iedb.org/population/). The analysis included global, Southeast Asian, and Philippine populations for MHC class I coverage. This assessment estimates the potential reach and effectiveness of the vaccine candidate within these populations (Immune Epitope Database, n.d.). The output provided: (1) projected population coverage, (2) the average number of epitope hits/HLA combinations recognized by the population, and (3) the minimum number of epitope hits/HLA combinations recognized by 90% of the population (Immune Epitope Database, n.d.).

### Molecular docking analysis of the candidate epitopes

To evaluate the structural and thermodynamic feasibility of epitope–HLA interactions, molecular docking was performed on the top five cytotoxic T lymphocyte (CTL) epitopes – selected based on the lowest IC_50_ values predicted using the IEDB MHC I binding tool – derived from the five *H. pylori* virulence factors. Each epitope was docked onto its corresponding HLA class I allele to assess binding orientation, interaction stability, and affinity.

Crystal structures of the selected HLA molecules were retrieved from the Protein Data Bank (PDB). Preprocessing involved removing heteroatoms, water molecules, and non-essential ligands to ensure optimal docking conditions, following the protocols described by Hernandez-Santoyo et al. (2013). Energy minimization and structural refinement of the HLA proteins were carried out using the GalaxyPepDock server (https://galaxy.seoklab.org/PEPDOCK), which supports flexible peptide docking and accurate modeling of peptide–HLA interactions. The server applies a template-based approach, utilizing the highest template modeling (TM) scores to guide structure alignment. Peptide–HLA complexes were modeled under flexible conditions to simulate conformational adjustments in both the peptide and the HLA binding groove. The resulting complexes were visualized and analyzed using UCSF ChimeraX v1.3, enabling the assessment of hydrogen bonding interactions, bond lengths, and spatial compatibility.

Binding affinities were quantified by calculating the Gibbs free energy change (ΔG) and dissociation constant (*K*_D_) at physiological temperature (37°C) using the PRODIGY web server (https://wenmr.science.uu.nl/prodigy/). These thermodynamic parameters were compared with reference HLA–epitope complex structures from curated databases to contextualize the predicted binding strength and stability. Peptides demonstrating the strongest and most stable binding to prevalent HLA alleles in the target populations were prioritized for downstream analysis.

## Results

### Highly conserved sequences from the selected virulence factors of H. pylori

Conservation analysis of the five selected *H. pylori* virulence proteins – urease, HP-NAP, OipA, HopZ, and SabA – revealed several regions with low sequence variability, as indicated by Shannon entropy values ≤ 0.1. Urease and HP-NAP showed the highest levels of conservation, with long conserved fragments ranging from 32 to 198 amino acids, consistent with their essential biological roles and evolutionary stability across diverse *H. pylori* strains. These findings align with previous studies highlighting the functional indispensability and sequence conservation of urease (Sarabi et al. 2025) and HP-NAP (Satin et al. 2000).

In contrast, conserved regions in OipA, HopZ, and SabA were shorter and more fragmented, ranging from 11 to 36 amino acids, suggesting these proteins may be under stronger selective pressure or subject to more frequent antigenic variation. Across all five proteins, a total of 27 conserved regions were identified, with an average of 5.4 conserved fragments per protein. Approximately 58% of the predicted CTL epitopes overlapped with these conserved regions, reinforcing their potential as stable immunogenic targets. A detailed summary of conserved fragments and corresponding epitope mapping is provided in the supplementary material.

### CTL epitopes mapped from the conserved sequences

CTL epitopes were selected in this study because they play a critical role in eliminating infected cells through specialized proteins, T-cell receptors (TCRs), present on their surface (Mulpuru and Mishra 2021). CTL epitopes were mapped from the conserved sequences of the five virulence factors, prioritizing candidates with positive transport-associated protein (TAP) scores, positive proteasome scores, and IC_50_ values ≤ 500 nM, as these parameters are crucial for MHC class I binding.

**Figure 2:**
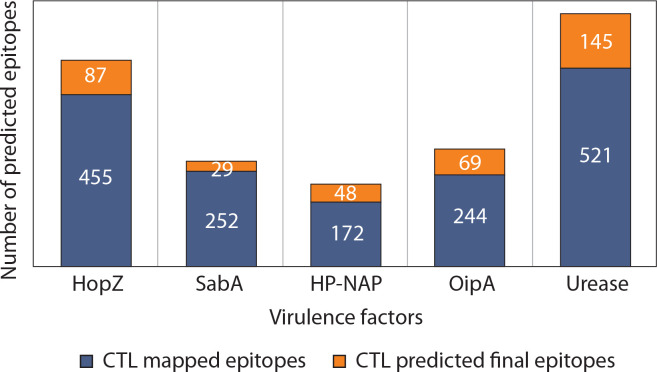
Comparative analysis of CTL epitope mapping and prediction across five major *Helicobacter pylori* virulence factors. The stacked bar chart illustrates the number of cytotoxic T lymphocyte (CTL) epitopes identified for each of the five virulence factors: HopZ, SabA, HP-NAP, OipA, and urease. Each bar is divided into two components: (i) CTL mapped epitopes (blue segment), representing the total number of epitopes identified using immunoinformatics tools based on binding affinity and immunogenicity, and (ii) CTL predicted final epitopes (orange segment), indicating the subset of mapped epitopes that passed stringent filters for antigenicity, non-allergenicity, non-toxicity, and population coverage criteria to qualify for final vaccine construct inclusion

The total number of candidate CTL epitopes per protein is summarized in Figure 2. Urease yielded the highest number of both mapped (*n* = 521) and final predicted CTL epitopes (*n* = 145), highlighting its strong immunogenic potential. In contrast, SabA had the lowest total epitope count, reflecting its comparatively limited CTL immunogenic profile. These results support the differential immunogenic roles of *H. pylori* virulence factors in epitope-based vaccine design. Detailed sequence information is provided in the Supplementary material.

### Population coverage of the CTL epitopes

In 2022, the global prevalence of *H. pylori* was 43.9% in adults and 35% in children and adolescents. While prevalence has generally declined in adult populations worldwide, it has not decreased in children and adolescents (Chen et al. 2024). In Southeast Asian countries, *H. pylori* prevalence ranges from 20% to 69%, contributing significantly to the incidence of peptic ulcer disease and gastric cancer (Quach et al. 2018). Among these countries, *H. pylori* remains the most critical risk factor for gastric cancer in the Philippines, with a prevalence of 34% in the general population as of 2018 (Quebral et al. 2022).

**Figure 3:**
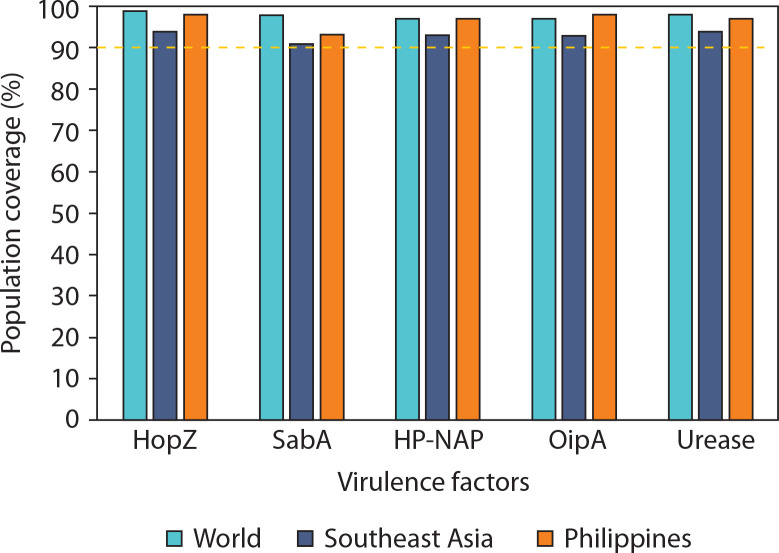
Predicted population coverage of T-cell epitopes derived from *Helicobacter pylori* virulence factors across different geographic regions. Bar chart representation of the estimated population coverage percentages for epitope-based vaccine candidates derived from five major *H. pylori* virulence factors – HopZ, SabA, HP-NAP, OipA, and urease – in three population groups: the global population (green bars), Southeast Asia (blue bars), and the Philippines (orange bars). The analysis was performed using the IEDB Population Coverage Tool based on HLA binding predictions and distribution data. A threshold of 90% population coverage (marked with a dashed line) was used to evaluate the potential broad utility of each antigen

Given the persistently high prevalence of ulcers and gastric cancer cases induced by *H. pylori* in these regions, determining the population coverage of selected virulence factors is essential to ensure optimal immune responses in vaccine formulation. All virulence factors achieved population coverage well above the threshold in all evaluated regions, with the lowest being SabA in Southeast Asia (91.39%), which still met the minimum coverage criterion (Figure 3). HopZ and urease exhibited the most consistent and highest population coverage globally, underscoring their strong immunogenic potential. These results support the selection of multiepitope vaccine constructs that provide high geographic inclusivity and immunogenetic relevance.

Moreover, this high value indicates that the epitopes could potentially bind to HLA molecules present in a large portion of the selected population (Bragais et al. 2025). Such high population coverage suggests a broader CTL response upon exposure to the epitopes (Longmate et al. 2001). Among the virulence factors, epitopes from HopZ had the highest predicted population coverage across all three selected regions, indicating the broadest CTL response and suggesting its potential to protect a larger proportion of individuals through vaccine development against *H. pylori*-induced diseases. Notably, the candidate epitope from HopZ with the highest coverage recorded 97.42% in the Philippines.

Other specific epitopes with the highest population coverage per locality for all virulence factors, along with their corresponding HLA class I alleles, are listed in the Supplementary material. Candidate epitopes generally showed high population coverage in the Philippines, which may be particularly significant given the country’s high *H. pylori* infection rates and the growing antibiotic resistance in the treatment of gastric and duodenal ulcers (Quebral et al. 2022). These high-coverage epitopes are predicted to be highly immunogenic, capable of eliciting strong immune responses and binding effectively to a wide range of MHC molecules present in the target population. Such epitopes are ideal candidates for inclusion in future vaccines, as they could potentially induce protective immunity in a substantial segment of the population (Cun et al. 2020).

### Assessment of potential toxicity, allergenicity, and cross-reactivity of CTL epitopes

CTL epitopes were further evaluated for potential toxicity, allergenicity, and cross-reactivity after UniProt analysis confirmed the absence of post-translational modifications in all five virulence factors. Except for urease, which contained 22 toxic epitopes, all other virulence factors yielded nontoxic candidate epitopes. However, all five proteins contained probable allergenic epitopes, reducing the final number of viable candidates.

Protein BLAST analysis identified cross-reactive epitopes with *E*-values lower than 1, with the lowest recorded *E*-value being 0.016 for SabA. Epitopes deemed noncross-reactive had *E*-values ranging from 1 to 271. The total number of final candidate CTL epitopes per virulence factor is presented in Figure 2. Overall, the results suggest that the top epitopes listed in the supplementary material are optimal candidates for vaccine formulation, as they are nontoxic, nonallergenic, noncross-reactive, and exhibit high population coverage.

### Molecular docking analysis of selected T-cell epitopes against HLAs

**Figure 4:**
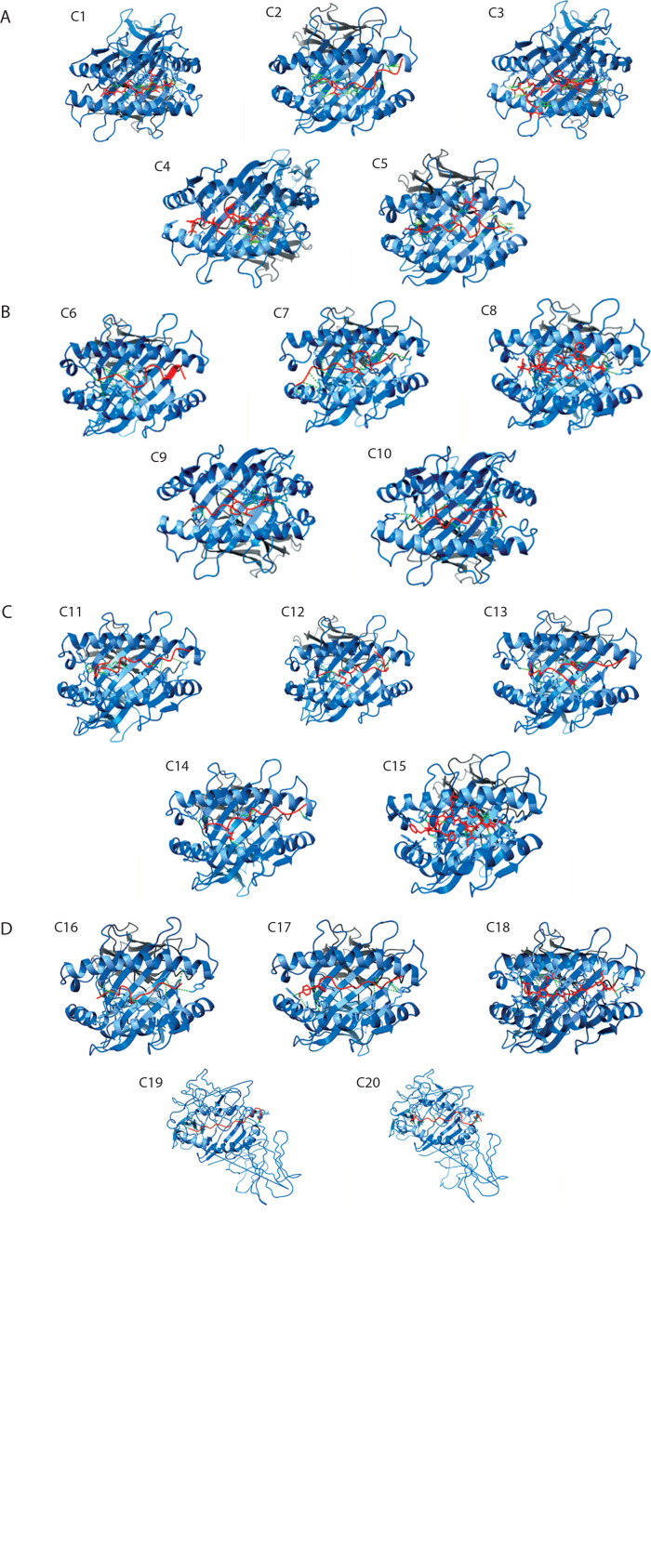
Molecular docking analysis of the top five predicted epitopes from the five virulence factors of *Helicobacter pylori* (**A** – HopZ; **B** – SabA; **C** – HPNAP; **D** – OipA; **E** – urease) showing the epitopes (colored in red) docked within the peptide binding groove of their corresponding HLAs with hydrogen bonds (green-dotted lines) as the main stabilizing intermolecular force

**Figure 5:**
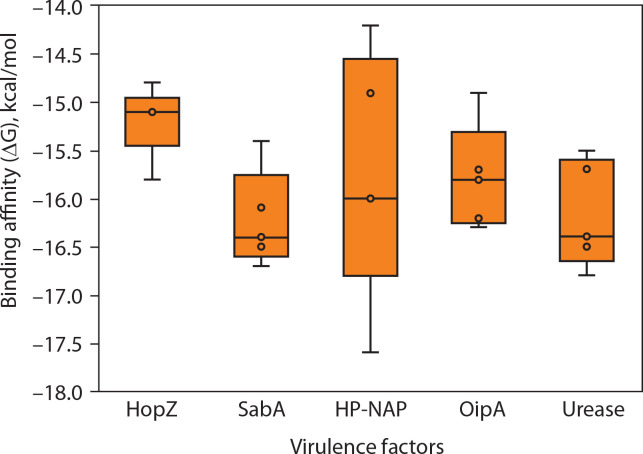
Predicted binding free energy (ΔG) of epitopes derived from *Helicobacter pylori* virulence factors. This panel presents the distribution of binding free energy (ΔG, in kcal/mol) values for epitopes originating from five *H. pylori* virulence factors: HopZ, SabA, HP-NAP, OipA, and urease. The ΔG values were obtained through molecular docking simulations, with more negative values indicating stronger predicted binding to MHC molecules. Among the evaluated factors, HopZ epitopes demonstrated the most favorable and consistent binding affinities, while HP-NAP epitopes exhibited the widest range and variability, reflecting diverse binding strengths. Each dot represents an individual epitope, with boxplots summari-zing the median, interquartile range, and full data spread

**Figure 6:**
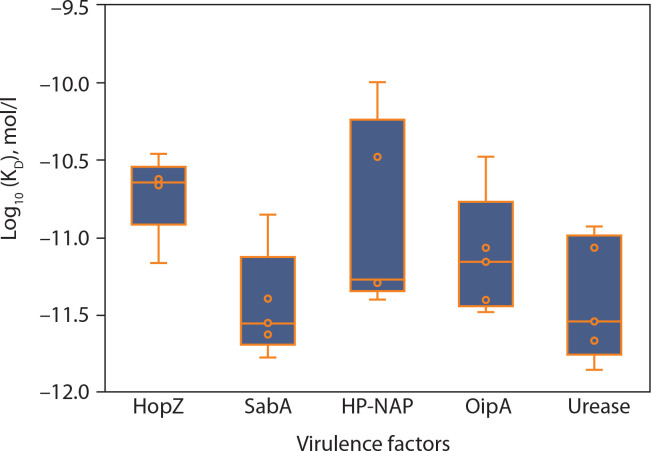
Predicted dissociation constant (*K*_D_) of epitopes from *Helicobacter pylori* virulence factors. This panel displays the log-transformed dissociation constant (log_10_ *K*_D_ in mol/l) for the same set of epitopes, providing insight into binding stability. Lower *K*_D_ values indicate tighter binding to MHC molecules. The overall trend aligns with the binding free energy data, with HopZ showing the most stable and uniform binding profiles, and HP-NAP again exhibiting a broader range of predicted affinities. Each point corresponds to a unique epitope, and the boxplots summarize the distribution of values across each virulence factor. These results support the prioritization of specific antigenic targets for vaccine design

Figure 4A–E shows the consolidated structures of the op five highest binding affinity based on IC_50_ values CTL epitopes docked within the peptide-binding groove of their corresponding HLA alleles. Favorable formation of HLA–epitope complexes was confirmed by negative ΔG values and low dissociation constants. All epitopes were classified as good to strong binders to their respective HLA alleles based on these ΔG and *K*_D_ values (Du et al., 2016). Specifically, negative ΔG values (Figure 5) indicate spontaneous binding, while low *K*_D_ values (Figure 6) correspond to higher binding affinity (Pan et al. 2016).

**Figure 7:**
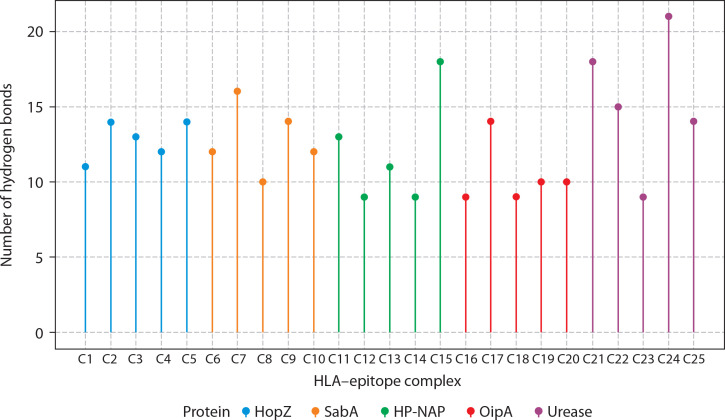
Hydrogen bond interactions between HLA molecules and predicted CTL epitopes from *Helicobacter pylori* virulence factors. The lollipop plot presents the number of hydrogen bonds formed in molecular docking simulations between selected HLA alleles and cytotoxic T lymphocyte (CTL) epitopes derived from five *H. pylori* virulence factors: HopZ (blue), SabA (orange), HP-NAP (green), OipA (red), and urease (purple). Each bar (C1–C25) corresponds to a distinct HLA-epitope complex. Hydrogen bonds, being key contributors to molecular stability and specificity, serve as a proxy for binding affinity and interaction strength. Notably, urease-derived epitopes (C21–C25) formed the highest number of hydrogen bonds (up to 21), suggesting robust and stable HLA interactions. In contrast, OipA epitopes showed comparatively fewer hydrogen bonds. These results support the structural feasibility and immunogenic promise of selected epitopes for downstream inclusion in multi-epitope vaccine constructs. Complexes were color-coded by their source protein to highlight intra-protein variability in HLA binding interactions

Additionally, 3D visualization (Figure 4A–E) of the HLA–epitope complexes revealed that most intermolecular interactions were hydrogen bonds between the HLA peptide-binding groove and the epitope. The importance of hydrogen bonds in stabilizing interactions between proteins (HLA alleles) and ligands (epitopes) is well-documented (Chen et al. 2016; Paul et al. 2013). Figure 7 presents the number of hydrogen bonds, while the corresponding donor–acceptor distance ranges are summarized in Table 1.

**Table 1:** Molecular docking analysis of protein–peptide interactions within HLA–epitope complexes across *Helicobacter pylori* virulence factors

Secretory protein	HLA–epitope complex	Donor–acceptor hydrogen distance range [Å]	Donor–acceptor distance range [Å]
HopZ	C1: HLA-A*02:06-ALNAAVGL	2.850–4.805	1.967–4.166
	C2: HLA-A*32:01-ALNAAVGLWNVI	2.906–4.603	1.925–3.936
	C3: HLA-A*02:06-FLFNLGLRM	2.881–4.999	1.899–4.296
	C4: HLA-B*40:02-FQFLFNLGL	2.757–5.142	1.907–3.709
	C5: HLA-B*40:02-FQFLFNLGLRMNL	2.763–4.434	1902–3.667
SabA	C6: HLA-A*02:06-AVITSVLGFWSL	2.872–5.076	1.918–4.061
	C7: HLA-A*02:06-GLFGGIQLAGTTWL	2.737–5.108	1.979–4.183
	C8: HLA-A*02:06-IQLAGTTWL	2.876–4.585	1.861–3.309
	C9: HLA-A*02:06-LRYYGFFDY	2.852–4.726	1.923–3.935
	C10: HLA-A*24:02-RLYSVYLNYV	2.701–3.699	1.940–2.970
HP-NAP	C11: HLA-A*02:06-AIVLFMKVHNFHWNV	2.796–5.055	1.977–4.103
	C12: HLA-A*02:06-HLQADAIVLFMKV	2.911–4.995	1.977–4.227
	C13: HLA-A*68:02-IVLFMKVHNFHWNV	2.832–4.962	1.898–4.233
	C14: HLA-A*23:01-LFMKVHNFHWNV	2.784–5.001	1.869–4.278
	C15: HLA-A*11:01-SFHSKDIFK	2.727–4.682	1.950–3.786
OipA	C16: HLA-A*02:06-FLPYGFNTDLL	2.881–4.703	2.712–3.694
	C17: HLA-A*02:06-FLPYGFNTDLLI	2.855–4.944	2.800–3.052
	C18: HLA-B*40:02-YQLGGVGSV	2.708–4.721	2.781–3.658
	C19: HLA-B*15:01-YQLGGVGSVPGSGL	2.867–4.750	2.722–3.328
	C20: HLA-B*15:01-YQLGGVGSVPGSGLI	2.839–5.642	2.666–3.232
Urease	C21: HLA-A*02:06-ATFNPELADFTIFV	2.791–5.203	1.881–4.430
	C22: HLA-A*02:06-ALTRHMSKDYDMAV	2.734–4.364	1.886–3.644
	C23: HLA-B*40:02-EEMHGRFPNLELL	3.064–5.152	2.155–4.380
	C24: HLA-B*40:02-EEMHGRFPNLELLL	2.723–4.953	1.868–4.030
	C25: HLA-A*02:06-DLLVINKIDLAPYV	2.801–4.839	1.966–4.031

This table summarizes the results of molecular docking simulations evaluating the interactions between predicted T-cell epitopes from *H. pylori* virulence factors and their corresponding human leukocyte antigen (HLA) molecules. Each entry represents a distinct HLA–epitope complex (C1–C25), categorized by the originating protein – HopZ, SabA, HP-NAP, OipA, or urease. The table includes the HLA allele and the associated epitope sequence, along with two key interaction metrics: the hydrogen bond donor–acceptor hydrogen distance range (Å) and the overall donor–acceptor distance range (Å). These values reflect the strength and stability of the peptide–HLA interaction, where shorter distances generally indicate stronger hydrogen bonding and more stable complex formation. Notably, most complexes exhibit hydrogen distances within an optimal binding range (2.7–5.1 Å), supporting the potential immunogenicity of these peptide candidates

## Discussion

*H. pylori* is uniquely adapted to survive in the acidic gastric environment through the production of urease and other virulence factors, which neutralize stomach acid and facilitate colonization of the gastric mucosa (Ali and AlHussaini 2024). This colonization impairs bicarbonate secretion and induces inflammation, increased acidity, and gastric metaplasia – pathological features associated with gastroduodenal diseases such as ulcers (Malik et al. 2023). Deeper penetration into the muscularis mucosa can lead to the formation of gastric lesions in both the stomach and duodenum, hallmark features of gastric and duodenal ulcers.

Because of the strong correlation between *H. pylori* infection and gastroduodenal ulceration, treatment typically combines antibiotics with PPIs (Ali and AlHussaini 2024). Current first-line regimens include concomitant therapy, sequential therapy, clarithromycin-based triple therapy, and bismuth quadruple therapy, all of which can contribute to improved gut microecology and overall gastrointestinal health (Li et al. 2023). By inhibiting the gastric proton pump, PPIs increase the bioavailability of acid-sensitive antibiotics and impair *H. pylori* metabolism and proliferation.

However, treatment efficacy is increasingly compromised by rising antibiotic resistance, recurrence of ulcers, high treatment costs, and adverse side effects – including an elevated risk of enteric infections, coronary complications, disruptions in magnesium and calcium homeostasis, and acute kidney injury (Ali and Al-Hussaini 2024).

Given these limitations, the development of a safe and effective vaccine remains a critical strategy for the longterm prevention of *H. pylori* infection. To date, no commercial vaccine is available, largely due to difficulties in identifying conserved antigenic targets and the extensive strain variability of *H. pylori* (Yunle et al. 2024). This gap highlights the potential of immunoinformatics as a powerful approach to address the limitations of traditional vaccine development.

By leveraging advanced computational tools, immunoinformatics enables high-throughput analysis of pathogen genomes and proteomes to identify and characterize immunodominant T-cell and B-cell epitopes (Arora et al. 2022; Chakraborty et al. 2021). This methodology has already been successfully applied to vaccine development against bacterial, viral, and parasitic pathogens (Keshri et al. 2023), significantly reducing development timelines from decades to just 2–3 years, while also providing a cost-effective alternative to conventional approaches (Chaleshtori et al. 2023; Chakraborty et al. 2021).

Both virulence factors and surface-exposed proteins are ideal vaccine targets due to their accessibility and immunogenic potential (Jebali et al. 2024). In this study, five *H. pylori* virulence proteins were selected: SabA and HopZ (involved in host adhesion), HP-NAP and OipA (linked to immune modulation), and urease (critical for acid stress survival). These proteins serve as antigenic determinants capable of being recognized by B cells, T cells, and antibodies, thereby eliciting robust immune responses and supporting their candidacy in vaccine design (Jebali et al. 2024; Kalali et al. 2014).

To enhance the efficacy of a multi-epitope vaccine against *H. pylori*, this study conducted a comprehensive immunoinformatics analysis of five key virulence-associated proteins. More than 100 full-length sequences for each protein were retrieved from the NCBI database to capture representative diversity across *H. pylori* strains. This broad, sequence-based approach enabled the identification of highly conserved regions, which are essential for overcoming the antigenic variability that has hindered the success of conventional vaccine strategies (Haghighi et al. 2024). Conserved epitopes are particularly valuable because they increase the likelihood of eliciting cross-protective immunity, thereby reducing the risk of immune escape – a major challenge when targeting genetically diverse pathogens (Meza et al. 2017).

Notably, high levels of conservation were observed in urease and HP-NAP, reflecting their indispensable roles in *H. pylori* pathogenesis. Urease plays a pivotal role in gastric acid neutralization, while HP-NAP functions as a neutrophil-activating protein that modulates host immune responses. The essential physiological functions of these proteins likely limit their tolerance to mutations, thereby stabilizing their epitope regions and reinforcing their suitability as vaccine targets. These findings are consistent with previous studies that have identified urease and HP-NAP as promising immunogens due to their evolutionary conservation and immunostimulatory properties (Pachathundikandi et al. 2016; Del Giudice et al. 2009).

The identification of conserved regions across all five virulence proteins substantially enhances the statistical confidence of epitope predictions by minimizing sampling bias and increasing predictive accuracy (Yurina 2020). By leveraging a comprehensive dataset and targeting functionally indispensable, evolutionarily conserved protein regions, this study addresses the critical challenge of antigenic variability while advancing the strategic design of a multiepitope vaccine. Such a vaccine has strong potential to elicit durable immunity, limit immune escape, and provide broad protection against diverse *H. pylori* strains, thereby overcoming a major barrier in combating this globally prevalent pathogen (Olawade et al. 2024).

To ensure robust cellular immune activation, CTL epitopes were predicted within the conserved domains. CTLs, presented via MHC class I molecules, mediate the direct elimination of infected epithelial cells (Peters et al. 2020). The identified epitopes demonstrated strong binding affinities (IC_50_ ≤ 500 nM) and promiscuous binding to a wide range of HLA alleles – a critical feature for achieving broad population coverage, particularly in ethnically diverse regions such as Southeast Asia and the Philippines, where *H. pylori* prevalence remains high.

Population coverage analysis using the IEDB tool predicted over 90% coverage both globally and regionally, aligning with WHO recommendations for vaccines targeting high-burden, resource-limited populations (WHO 2017; Kaur et al. 2023). In the Philippines, predicted coverage ranged from 93.14% (SabA) to 97.55% (HopZ), while in Southeast Asia, it ranged from 91.39% (SabA) to 94.13% (HopZ). Globally, coverage ranged from 97.28% for OipA to 98.85% for HopZ. These values underscore the regional relevance, potential efficacy, and universal applicability of the proposed vaccine candidates, offering promising protection across diverse human populations.

The population coverage values reported in this study align with accepted benchmarks for effective vaccine design, wherein an ideal candidate should encompass at least 90% of a population’s HLA allele distribution to ensure broad immunogenicity across diverse ethnic groups (Sarvmeili et al. 2024). Notably, all five virulence factors examined – particularly HopZ, SabA, and urease – surpassed this threshold both regionally and globally, each achieving coverage rates exceeding 97%. These findings underscore the strong potential of the proposed multiepitope vaccine to elicit effective immune responses across genetically diverse populations (Bragais et al. 2025). Such high coverage is especially important for *H. pylori*, a pathogen known for its high genetic variability and immune evasion capabilities.

While the predictions suggest broad applicability, it is important to acknowledge that HLA allele distributions vary across ethnic and geographic populations. Therefore, future studies should prioritize population-specific immunogenicity assessments and clinical validation to confirm efficacy in regions with differing HLA profiles. Moreover, the high HLA-binding promiscuity of the predicted epitopes enhances their potential for universal application. Promiscuous epitopes can bind to multiple HLA alleles, making them suitable for diverse genetic backgrounds and increasing the likelihood of effective antigen presentation (Frahm et al. 2007). The predicted epitopes in this study demonstrated strong binding affinities (IC_50_ ≤ 500 nM), further reinforcing their immunogenic potential.

In addition to MHC-binding predictions, other key immunological parameters were evaluated. Positive TAP scores indicate efficient peptide transport into the endoplasmic reticulum, a critical step in MHC class I antigen presentation. Similarly, favorable proteasome scores suggest optimal proteolytic cleavage, facilitating the generation of peptides suitable for MHC presentation (Lehnert and Tampé 2017). Together, these properties ensure that the selected epitopes are efficiently processed and recognized by CTLs.

The central role of MHC molecules in antigen presentation further underscores the importance of binding promiscuity. T-cell receptors (TCRs) on CTLs can only recognize antigenic peptides when presented by MHC molecules, which are encoded by highly polymorphic genes in vertebrates. These genes help distinguish self from non-self, forming the foundation of adaptive immune recognition (Najafian and Riella 2019). Consequently, integrating MHC-binding analyses into vaccine design is fundamental to achieving both efficacy and population-wide applicability.

Beyond MHC binding affinity, additional antigenprocessing parameters – specifically TAP transport and proteasome cleavage scores – were incorporated into epitope prioritization to enhance predictive accuracy. A positive TAP score reflects efficient transport of peptides into the endoplasmic reticulum for MHC class I loading, whereas a high proteasome score indicates favorable C-terminal cleavage, both of which are essential for effective antigen presentation and subsequent CTL activation (Doytchinova et al. 2004; Lehnert and Tampé 2017).

Epitopes with IC_50_ values ≤ 500 nM were prioritized, as they are predicted to exhibit strong binding to MHC class I molecules and are more likely to be naturally processed and presented on the cell surface (Paul et al. 2013). Incorporating these parameters strengthens the biological plausibility of the predicted epitopes, ensuring their immunological relevance *in vivo* and improving the translational potential of the *in silico* findings.

This study primarily focused on MHC class I-restricted epitopes, which play a pivotal role in activating CD8^+^ cytotoxic T cells and driving cell-mediated immunity against intracellular pathogens such as *H. pylori*. However, the exclusion of MHC class II-restricted epitopes and post-translational modification (PTM) sites represents a limitation, as helper T-cell (Th) activation is critical for orchestrating long-term immunity, including memory responses and B-cell maturation. Future analyses will incorporate MHC class II predictions to provide a more comprehensive immune profile and facilitate the development of a vaccine capable of inducing both humoral and cellular immune responses.

Among the five virulence proteins analyzed, urease generated the highest number of predicted CTL epitopes, underscoring its strong immunogenic potential. The abundance of MHC class I-compatible epitopes supports urease’s candidacy as a key component in a multi-epitope vaccine formulation. Conversely, HP-NAP yielded fewer CTL-mapped epitopes, potentially reflecting structural or antigen-processing constraints. Nonetheless, its high conservation and established adjuvant-like properties justify its inclusion as both an immune enhancer and a potential target for CD4^+^ helper T-cell responses. This is consistent with prior findings demonstrating that HP-NAP promotes Th1-skewed immune responses, thereby enhancing its utility in vaccine formulations aimed at intracellular pathogens (Del Giudice et al. 2009).

In addition to achieving broad immunogenicity through effective MHC binding, ensuring the safety of vaccine candidates is a critical component of epitope-based vaccine design. To address this, a multitiered *in silico* screening pipeline was employed to assess toxicity, allergenicity, and human cross-reactivity. Toxic epitopes were identified using support vector machine (SVM)-based predictive models, which analyze physicochemical features associated with cytotoxic potential. Epitopes predicted as toxic were systematically excluded to prevent unintended inflammatory or cytolytic effects in host tissues. This approach aligns with previous findings indicating that nontoxic peptides are more likely to elicit protective immunity with minimal adverse reactions (Arya and Bhatt 2021). Notably, 22 toxic epitopes predicted within the urease protein were removed to enhance the safety and tolerability of the final vaccine construct.

Allergenicity screening further refined the epitope pool. Using allergen databases and structural similarity metrics, such as Tanimoto coefficients, epitopes with high similarity to known allergens were excluded. This conservative filtering step minimizes the risk of IgE-mediated hypersensitivity reactions, which could compromise both vaccine efficacy and public acceptance. Prior studies have emphasized that even lowfrequency allergenic responses can negatively affect the success of multiepitope vaccines (Rencilin et al. 2021; Prawiningrum et al. 2022). All five virulence factors initially contained potential allergenic epitopes, which were subsequently eliminated, ensuring that only nonallergenic candidates were retained for further development.

To mitigate the risk of autoimmune responses, cross-reactivity screening against the human proteome was conducted. Epitope sequences were analyzed using BLASTp, and only those with *E*-values greater than 1 – indicating low homology to human proteins – were retained. This step significantly reduces the likelihood of molecular mimicry and ensures specificity to *H. pylori*-derived antigens (Pahari et al. 2017). Such specificity is critical not only for avoiding immune tolerance and autoimmunity but also for enhancing the diagnostic potential of the identified epitopes by ensuring that immune responses are unique to *H. pylori*.

Collectively, these safety assessments underscore the importance of integrating both immunogenic and safety parameters in the rational design of epitope-based vaccines. The exclusion of toxic, allergenic, and cross-reactive epitopes substantially strengthens the translational viability of the candidate vaccine and aligns with best practices in next-generation vaccine development. These findings are consistent with emerging literature advocating for preclinical computational filtering as a cost-effective and reliable strategy to enhance vaccine safety and maximize public health impact (Bragais et al. 2025; Ysrafil et al. 2022; Jebali et al. 2024).

Molecular docking simulations between *H. pylori* epitopes and HLA alleles yielded critical binding metrics, including dissociation constants (*K*_D_) and Gibbs free energy (ΔG), as summarized in Figures 5 and 6. The predicted *K*_D_ values ranged from 10^–10^ to 10^–12^ M, reflecting high-affinity interactions between the epitopes and the peptide-binding grooves of HLA molecules. These low dissociation constants indicate the formation of stable complexes under physiological conditions – a key prerequisite for effective antigen presentation and T-cell activation, both essential for initiating cytotoxic immune responses against infected cells (Qin et al. 2017; Weber et al. 2009).

Consistent with these findings, all HLA–epitope complexes exhibited negative ΔG values, indicating thermodynamically favorable interactions. Negative Gibbs free energy reflects spontaneous complex formation, driven by favorable enthalpic and entropic contributions (Kamran 2021; Barazesh et al. 2024). The dual observation of low *K*_D_ values and negative ΔG supports both the structural compatibility and immunological relevance of the selected epitopes, reinforcing their candidacy for inclusion in a multiepitope vaccine construct targeting *H. pylori*-associated pathologies, including gastritis, peptic ulcers, and gastric cancer.

Beyond affinity and thermodynamics, docking simulations also evaluated hydrogen-bonding interactions, which are critical for stabilizing epitope–HLA complexes. Donor–acceptor distances were measured to assess the quality of these hydrogen bonds, which play a vital role in epitope anchoring, exclusion of water molecules from the binding interface, and overall complex integrity (Chen et al. 2016). As detailed in Table 1, the observed donor–acceptor distances ranged from 1.861 Å to 4.430 Å. According to established structural models, hydrogen bonds within 2.2–2.5 Å are classified as strong (often with partial covalent character), those within 2.5–3.2 Å are considered moderate (electrostatic), and those exceeding 3.2 Å are generally weak, contributing minimally to binding stability (Jeffrey 1998; McRee 1999).

This range of interaction distances indicates variability in peptide–HLA complex quality. Notably, certain complexes – such as HLA-A*02:06 bound to the epitope FLPYGFNTDLLI – demonstrated donor–acceptor distances within the optimal 2.2–3.2 Å range, suggesting structurally favorable and potentially immunodominant interactions. Such geometrically optimal hydrogen bonds increase the likelihood of stable immune recognition and effective T-cell engagement.

Taken together, the molecular docking results demonstrate that epitopes capable of forming high-affinity, thermodynamically favorable, and structurally stable interactions with HLA alleles are prime candidates for vaccine development. By prioritizing epitopes that meet these combined criteria – particularly those forming hydrogen bonds within the ideal geometric range – subunit vaccine constructs can be rationally optimized to elicit robust immunogenicity while minimizing off-target effects. These findings provide strong computational support for the downstream development of epitope-based vaccines targeting *H. pylori* and its associated disease manifestations.

### Limitations of the study

This study’s integrated application of immunoinformatics tools provides a high-throughput and rational framework for the *in silico* identification and evaluation of vaccine targets – a methodology increasingly valued for expediting early-stage vaccine development (Ysrafil et al. 2022). While the approach offers a robust foundation for designing a multiepitope vaccine against *H. pylori*, several limitations should be acknowledged to ensure a balanced interpretation of the findings.

First, the current analysis focused exclusively on MHC class I-restricted epitopes, which primarily activate CTLs. The omission of MHC class II epitope prediction represents a significant limitation, as helper T-cell responses are essential for sustaining long-term immune memory, coordinating broader immune responses, and enhancing vaccine efficacy (Testa and Philip 2012). Including MHC class II predictions in future work will be critical for developing a more comprehensive and immunologically robust vaccine candidate.

Second, the study did not evaluate or quantify the potential impact of excluding epitopes that overlapped with predicted PTM sites. While this conservative approach was intended to maximize epitope stability and immunogenic consistency, it may have inadvertently removed immunologically relevant sequences, potentially reducing the overall diversity and coverage of the selected epitopes.

Third, while computational tools provide a powerful high-throughput platform for vaccine design, their predictive capabilities are inherently constrained by algorithmic limitations and assumptions. *In silico* tools can yield false positives, may not fully capture the complex dynamics of antigen processing and presentation, and are subject to biases in HLA allele databases (Gowthaman and Agrewala 2008). For example, certain HLA alleles are overrepresented in public datasets, which may skew epitope predictions and limit the generalizability of the findings to underrepresented populations.

Additionally, the reliance on predicted binding affinity (e.g., IC_50_ values, docking scores) as a proxy for immunogenicity may not fully reflect the biological reality of epitope recognition, processing, and presentation *in vivo*. As such, the computational findings reported here should be interpreted as hypotheses that require validation through laboratory-based immunological assays and animal models.

Finally, although this study prioritized safety and immunogenicity through *in silico* screening for toxicity, allergenicity, and human homology, these predictions cannot substitute for empirical safety assessments. Experimental approaches will be necessary to confirm the tolerability, immunogenicity, and protective efficacy of the proposed multiepitope vaccine.

## Conclusions

This study identified highly conserved sequences from five *H. pylori* virulence factors – HP-NAP, OipA, SabA, HopZ, and urease. All selected virulence factors achieved population coverage greater than 90%, indicating strong CTL response potential across most populations. Among these, HopZ demonstrated the highest predicted population coverage across all three evaluated regions, suggesting the broadest CTL response and the greatest potential efficacy for vaccine development against *H. pylori*-induced diseases.

Comprehensive *in silico* safety assessments, including cross-reactivity, allergenicity, and toxicity analyses, confirmed that all selected epitopes are nonreactive with human proteins, thereby reducing the likelihood of autoimmune responses. The identified epitopes also displayed high immunogenicity and strong binding affinity to MHC molecules, reinforcing their suitability for inclusion in multiepitope vaccine formulations. In future studies, it is recommended that further steps, such as molecular docking, be employed to refine epitope selection and finalize vaccine formulation. This would help ensure precise epitope–MHC interactions, optimize immune recognition, and ultimately contribute to the development of safe and effective vaccines against *H. pylori*, addressing the pressing global health challenge presented by this bacterium.

## Supplementary Material



## Future directions

Building on the current findings, future work should focus on the rational development of a full-length, multiepitope subunit vaccine incorporating the prioritized immunogenic regions identified through computational analyses. This effort should include the strategic integration of suitable adjuvants, linker sequences, and delivery platforms to collectively enhance immunogenicity, antigen stability, and appropriate immune activation. Codon optimization and *in silico* cloning into compatible expression vectors will facilitate efficient downstream synthesis and expression of the vaccine construct.

Structural modeling and molecular dynamics simulations should be employed to evaluate the vaccine’s conformational stability, epitope exposure, and interaction potential with immune receptors, thereby refining candidate selection prior to experimental testing. Once finalized, the construct must undergo comprehensive experimental validation, including antigen expression profiling, *in vitro* immunogenicity assays using human immune cells, and *in vivo* testing in relevant animal models to assess safety, immunogenicity, and protective efficacy.

In addition, integrating systems biology approaches – such as transcriptomic profiling – may provide deeper insights into host immune responses and guide further optimization of vaccine formulations for specific populations. While computational immunoinformatics offers a strong foundation for antigen selection, it is essential to validate these predictions through laboratory assays. Techniques such as cytokine release analysis, T-cell proliferation assays, and MHC–peptide binding assays will be instrumental in confirming functional immune responses.

Ultimately, advancing this multiepitope vaccine strategy from the computational stage toward clinical application will require a synergistic combination of *in silico* tools and experimental validation. This approach aligns with the growing shift toward subunit-based vaccines, which offer improved safety, scalability, and precision in targeting conserved and immunodominant regions of pathogens.
